# Alarming levels of fluoroquinolone resistance among MDR-TB patients in Poland: molecular and phenotypic analysis

**DOI:** 10.3389/fcimb.2026.1881444

**Published:** 2026-07-01

**Authors:** Monika Kozińska, Agnieszka Głogowska, Alina Minias, Jarosław Dziadek, Ewa Augustynowicz-Kopeć

**Affiliations:** 1 Department of Microbiology, National Tuberculosis and Lung Diseases Research Institute, Warsaw, Poland; 2 Laboratory of Genetics and Physiology of Mycobacterium, Institute of Medical Biology, Polish Academy of Sciences, Lodz, Poland

**Keywords:** fluoroquinolone, MDR-TB, tuberculosis, drug resistance, tNGS, gene mutation, Beijing genotype

## Abstract

**Background:**

Fluoroquinolones (FQs) are a cornerstone of modern multidrug-resistant tuberculosis (MDR-TB) treatment, particularly within the WHO-recommended all-oral BPaLM regimen. The emergence of FQ-resistant *Mycobacterium tuberculosis* (MTB) poses a critical threat to these life-saving therapies. While the global proportion of FQ resistance among MDR/RR-TB cases is estimated at 19%, phenotypic and molecular data from low-incidence countries in Central and Eastern Europe remain limited. This study aimed to comprehensively assess the FQ resistance profile of MDR/RR-TB strains isolated in Poland between 2022 and 2024.

**Methods:**

We analyzed 240 drug-resistant MTB isolates (228 MDR and 12 rifampicin-resistant) collected nationwide. Phenotypic drug susceptibility testing (DST) was performed using the Bactec MGIT 960 system. Isolates demonstrating resistance to levofloxacin and/or moxifloxacin (*n* = 104) were further analyzed using Deeplex-Myc TB targeted next-generation sequencing (tNGS) to identify mutations within the quinolone resistance-determining region (QRDR) and determine strain spoligotypes.

**Results:**

Phenotypic FQ resistance was detected in an alarming 43.0% (104/240) of isolates. Mutational analysis revealed eight variants in the *gyrA* gene and two variants in the *gyrB* gene. The most frequent mutations were D94G (44.2%), D94N (18.3%), and D94A (12.5%). Genome sequencing allowed the identification of 11 molecular families (spoligotypes), with the Beijing genotype being by far the dominant one and accounting for 91.0% of all isolates.

**Conclusions:**

Our findings demonstrate a substantial and concerning burden of FQ resistance among MDR-TB strains in Poland, significantly exceeding global averages. The high prevalence of QRDR mutations and the dominance of the Beijing genotype highlight the urgent need for systematic molecular surveillance and rapid NGS-based diagnostics. Such measures are essential to ensure the effectiveness of the BPaLM regimen and prevent the further spread of highly drug-resistant TB.

## Introduction

Tuberculosis (TB) is a major global health challenge, and its prevalence has increased over the past three years. According to the World Health Organization (WHO) Global Tuberculosis Report, in 2023, the estimated number of new cases was 10.8 million, with 400,000 patients diagnosed with multidrug-resistant tuberculosis (MDR-TB) (World Health Organization, 2024).

MDR strains are characterised by resistance to both rifampicin and isoniazid. Those with additional resistance to fluoroquinolones (FQs) are classified as pre-extensively drug-resistant (pre-XDR), while those showing additional resistance to at least one of the group A drugs (bedaquiline and/or linezolid) are identified as extensively drug-resistant (XDR) (Viney et al., 2021).

Treatment of II-line drug-resistant TB requires better resources and more financial support, so MDR-TB cases may be more prevalent in low-income communities where these conditions are difficult to meet (Gandhi et al., 2010).

Currently, the treatment regimen recommended for patients with MDR-TB and patients with rifampicin-resistant TB (RR-TB) is all-oral, 6-month treatment with bedaquiline, pretomanid and linezolid, in combination with FQ (BPaLM, bedaquiline–pretomanid–linezolid–moxifloxacin). This regimen demonstrates high efficacy, lower treatment costs and improved quality of life over the course of the disease (World Health Organization, 2022). FQs are synthetic chemotherapeutic agents with broad antimicrobial activity, including antimicrobial activity. Given their excellent pharmacokinetic and pharmacodynamic properties, they are considered the most effective II-line drugs for the treatment of MDR-TB (Dorman et al., 2021).

In many resource-limited countries where FQs are readily available as over-the-counter drugs, the problem of their abuse and misuse has emerged (Chen et al., 2011). The result of this phenomenon is the isolation of *Mycobacterium tuberculosis* (MTB) strains that are resistant to FQ, both mono-resistant and in combination with I-line drug resistance (Kim et al., 2025). Other causes of FQ resistance include incomplete adherence to the TB treatment regimen, failure of previous treatment and transmission through contact with patients with drug-resistant TB (Chang et al., 2010). According to the WHO 2023 report, the global proportion of MDR/RR-TB with resistance to any of the FQs was 19%. However, the scale of the phenomenon is not fully understood and varies between regions (Mayer and Takiff, 2014; Bernard et al., 2015; Bernard et al., 2016; World Health Organization, 2023a).

With the increasing role of FQs in the treatment of MDR/RR-TB, rapid detection of mycobacterial resistance to this drug group is crucial. This resistance is most often associated with mutations in the so-called quinolone resistance determinant region (QRDR) covering the *gyrA* and *gyrB* genes, leading to reduced efficacy of therapeutic regimens containing this FQ (World Health Organization, 2021). Despite the availability of global data, information on the phenotypic and molecular prevalence of FQ resistance in countries with low TB incidence, including Central and Eastern Europe, remains limited (European Centre for Disease Prevention and Control and WHO Regional Office for Europe, 2024). The lack of such data has important clinical and epidemiological implications in the context of current WHO recommendations for short, all-oral FQ-based MDR-TB treatment regimens.

The aim of this study was to comprehensively assess the phenotypic and molecular FQ resistance of MTB strains with MDR and RR resistance isolated in Poland between 2022 and 2024. The need for such an analysis was dictated, among other things, by the Russian-Ukrainian conflict, the reception of war refugees from Ukraine and, consequently, the increase in the number of foreigners in Poland with MDR-TB (Kozińska et al., 2025).

Using phenotypic and molecular diagnostic methods, the prevalence of FQ resistance in the patient population studied was assessed, the molecular basis of this resistance was identified, and spoligotypes of strains were identified.

## Materials

### Patients and bacterial isolates

The initial study group consisted of 240 patients with drug-resistant TB (228 with MDR-TB and 12 patients with RR-TB), confirmed by bacteriological testing between 2022 and 2024. The sample selected for the study represented 2% of all patients with culture-confirmed TB in Poland during this period (*N* = 11 684) and 80% of patients with MDR-TB (*N* = 301) (Korzeniewska-Koseła, 2025).

The missing 20% (*n* = 61) MDR/RR isolates corresponded to strains that regional laboratories did not provide, as part of the mandatory verification of drug-resistant strains, to the Department of Microbiology of the National Reference Laboratory for Mycobacteria (NRLM), or those that the NRLM was unable to revive or analyze molecularly due to poor viability.

**Figure 1:**
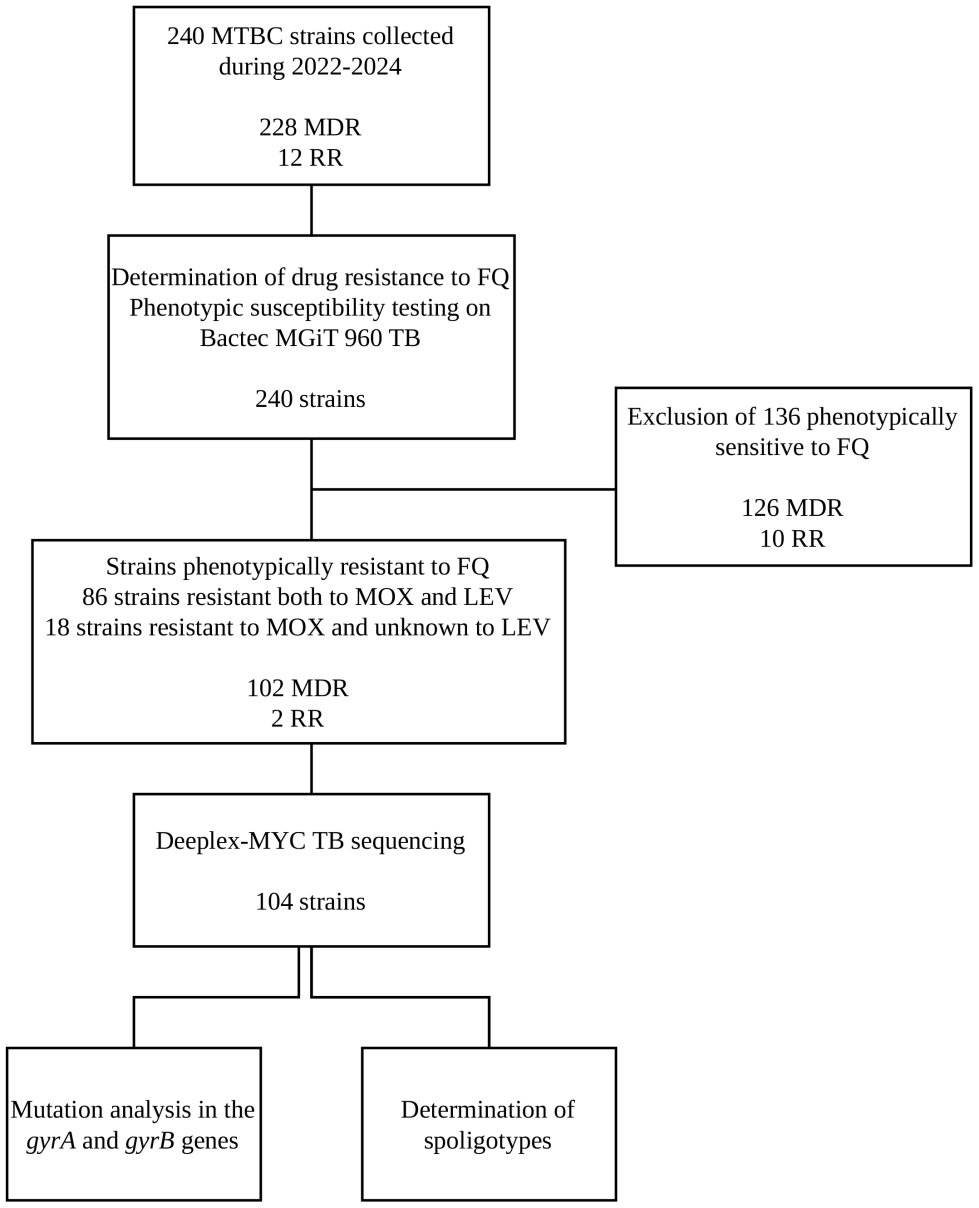
Study flowchart. The final dataset consists of 104 strains.

Following phenotypic verification of primary ((isoniazid (INH) and rifampicin (RIF))) and secondary (FQ) drug resistance, 104 strains with FQ resistance were selected for the sequencing stage (102 strains with MDR+FQ resistance and 2 strains with RR+FQ resistance) (**Figure 1**).

The selected strains (*n* = 104) were cultured from 81 men (78.0%) and 23 women (12.0%). Fifty six patients (54.0%) were foreigners, and 48 (46.0%) were of Polish nationality. The age of the patients ranged from 1 to 88 years.

### Methods

The initial identification of strains and the phenotype of resistance to basic antimycobacterial drugs were determined at regional mycobacterial laboratories, afterwards the strains were sent to the Department of Microbiology of the NRLM in Warsaw to verify the drug resistance profile and identify the spoligotype.

### Phenotypic drug resistance

Conventional drug susceptibility testing (DST) was performed using the Bactec MGIT 960 system (Becton Dickinson Diagnostic Systems, Sparks, MD, USA).

According to WHO guidelines, the critical concentrations for individual drugs were: INH, 0.10 μg/mL; and RIF, 1.00 μg/mL. To evaluate FQ resistance and distinguish between low-level and high-level resistance, moxifloxacin (MOX) was interpreted using two distinct thresholds: a critical concentration of 0.25 μg/mL defined standard (low-level) resistance, while a concentration of 1.00 μg/mL was utilized to determine high-level resistance (World Health Organization, 2018). Levofloxacin (LEV) susceptibility was assessed at a single critical concentration of 1.00 μg/mL.

### Genome sequencing

Genomic DNA was extracted from MTB strains using the Maxwell^®^CSC Pathogen Total Nucleic Acid Kit automated system (Promega, Madison, Wisconsin, USA). DNA concentration and purity (A260/A280) were assessed using a NanoDrop 2000 (Thermo Fisher Scientific, Waltham, MA, USA). Isolates were stored at -20 °C until use.

Genome sequencing of the selected 104 strains was performed on the Deeplex-Myc TB system (Genoscreen, Lille, France), according to the manufacturer’s procedure. Deeplex-Myc TB is an NGS-based assay for testing resistance to drugs used in the treatment of TB, as well as for identifying (sub)lines and spoligotypes of the *Mycobacterium tuberculosis* complex (Jouet et al., 2021).

## Results

### Phenotypic resistance

Resistance verification of 240 strains allowed 136 (57.0%) FQ-sensitive strains to be excluded from further analysis. The remaining 104 (43.0%) strains were characterized by phenotypic resistance to MOX and/or LEV (**Table 1**). All 104 strains showed phenotypic resistance to MOX. Phenotypic resistance to LEV was evaluated in 86 strains, as LEV testing was routinely introduced into diagnostics in 2023; consequently, 18 strains isolated in 2022 lacked LEV data. Among the 86 strains tested for LEV, 83 (96.5%) were resistant and 3 (3.5%) were susceptible.

**Table 1:** Phenotypic drug susceptibility profiles of *Mycobacterium tuberculosis* isolates stratified by specific target-site mutations within the QRDR region.

Genotype		Phenotype				
Gene/profile	Mutation/variant	LEV			MOX	
		Sensitive (N = 3)	Resistant (N = 83)	Missing data (N = 18)^*^	Sensitive (N = 0)	Resistant (N = 104)
*gyrA* (single)	G88C	0 (0.0%)	1 (1.2%)	0 (0.0%)	–	1 (1.0%)
	A90V	3 (100.0%)	5 (6.0%)	0 (0.0%)	–	8 (7.7%)
	S91P	0 (0.0%)	11 (13.3%)	0 (0.0%)	–	11 (10.6%)
	D94A	0 (0.0%)	13 (15.7%)	0 (0.0%)	–	13 (12.5%)
	D94G	0 (0.0%)	45 (54.2%)	1 (5.6%)	–	46 (44.2%)
	D94N	0 (0.0%)	19 (22.9%)	0 (0.0%)	–	19 (18.3%)
	D94Y	0 (0.0%)	3 (3.6%)	0 (0.0%)	–	3 (2.9%)
*gyrB* (single)	N499T	0 (0.0%)	1 (1.2%)	0 (0.0%)	–	1 (1.0%)
	E501V	0 (0.0%)	1 (1.2%)	0 (0.0%)	–	1 (1.0%)
Double Mutants	Within *gyrA* exclusively	0 (0.0%)	3 (3.6%)^a^	0 (0.0%)	–	3 (2.9%)^a^
Double Mutants	Combined *gyrA*+*gyrB*	0 (0.0%)	1 (1.2%)^b^	0 (0.0%)	–	1 (1.0%)^b^
Polymorphism	S95T (Global frequency)	3 (100.0%)	80 (96.4%)	18 (100.0%)	–	101 (97.1%)
Wild-type-like	S95T only (Lacking clinically significant)	0 (0.0%)	4 (4.8%)^c^	1 (5.6%)^c^	–	5 (4.8%)^c^

*Data from 18 isolates from 2022 are missing for the LEV phenotype due to diagnostic protocol changes.

^a^ Includes 3 isolates: 108/24 (S91P + D94N), 222 (A90V + D94N), and 142/24 (S91P + D94G.

^b^ Represents 1 isolate (352/23) harboring a combined gyrA D94G + gyrB E501V mutational profile.

^c^ Out of 5 MOX-resistant isolates carrying only the S95T polymorphism without additional resistance conferring mutations, 4 were phenotypically resistant to LEV, and 1 lacked LEV data.

### Analysis of mutations in the *gyrA* and *gyrB* genes

Phenotypically FQ-resistant strains were analyzed using the Deeplex-Myc TB system. Across the 104 strains, eight mutations in the *gyrA* gene (G88C, A90V, S91P, D94A, D94G, D94N, D94Y, S95T) and two mutations in the *gyrB* gene (N499T and E501V) were identified. The gyrA S95T substitution was detected in 101 (97.1%) strains. Excluding this S95T polymorphism, which is considered clinically insignificant for FQ resistance, 95 (91.0%) strains harbored a single resistance-conferring mutation within the QRDR region, while 4 (4.0%) strains carried double mutations. Among these double mutants, three involved exclusively the *gyrA* gene: isolate 108/24 (S91P + D94N), isolate 222 (A90V + D94N), and isolate 142/24 (S91P + D94G). The remaining double mutant involved a combination of both the *gyrA* and *gyrB* genes (isolate 352/23, carrying gyrA D94G + gyrB E501V). In 5 (4.8%) strains, only the S95T substitution was detected, with no additional mutations in the *gyrA* and *gyrB* resistance-determining regions.

The distribution of these genetic determinants varied distinctly across drug phenotypes (**Table 1**). Among the 104 MOX-resistant strains, the most prevalent resistance-conferring mutation was gyrA D94G, identified in 46 (44.2%) isolates, followed by D94N in 19 (18.3%), D94A in 13 (12.5%), S91P in 11 (10.6%), A90V in 8 (7.7%), D94Y in 3 (2.9%), and G88C in 1 (1.0%) isolate. Mutations in the *gyrB* gene were identified in 2 (2.0%) strains: one carried the N499T substitution and the other harbored the E501V change. Both *gyrB* mutations were accompanied by the S95T polymorphism, and the E501V mutant also represented the previously mentioned double mutant carrying a concurrent gyrA D94G mutation (isolate 352/23).

For LEV, phenotypic susceptibility was evaluated in 86 strains, as LEV testing was introduced into routine diagnostics in 2023, leaving 18 strains from 2022 without LEV data. Among the 86 tested strains, 83 (96.5%) were resistant and 3 (3.5%) were susceptible. In the LEV-susceptible group, all 3 strains (100%) carried the gyrA A90V mutation (alongside S95T). In contrast, this specific mutation was detected in 5 out of the 83 (6.0%) LEV-resistant strains. For the remaining mutations among the 83 LEV-resistant isolates, the frequencies were as follows: D94G was found in 45 (54.2%) isolates, D94N in 19 (22.9%), D94A in 13 (15.7%), S91P in 11 (13.3%), D94Y in 3 (3.6%), and G88C in 1 (1.2%) isolate. Regarding *gyrB*, the N499T mutation was present in 1 (1.2%) LEV-resistant strain, while the E501V mutation was identified in 1 (1.2%) strain.

Of the 5 MOX-resistant isolates that lacked additional resistance-conferring mutations (carrying only S95T), 4 were phenotypically resistant to LEV (representing 4.8% of the LEV-resistant cohort), and 1 lacked LEV data. A comprehensive summary of mutation frequencies stratified by drug phenotype is presented in **Table 1**, while the detailed isolate-level profiles have been moved to **Supplementary Table 1**.

### Spoligotyping and lineage distribution

Based on the analysis using the Deeplex-MYC TB sequencing system, the 104 phenotypically FQ-resistant MTB isolates were assigned to 11 molecular families (Spoligotype International Types, SITs) belonging to two major lineages: Lineage 2 (East-Asian) and Lineage 4 (Euro-American) (**Table 2**).

**Table 2:** Diversity of spoligotypes of 104 strains resistant to fluoroquinolones, included in the study.

Spoligotyping					No. of isolates (N=104)		Foreign-born (n=56)	Polish-born (n=48)
Lineage	Clade	Family	SIT*	Octal code				
Lineage 2 EAST-ASIAN	BEIJING	BEIJING	1	000000000003771	58 (56.0%)	95 (91.0%)	52 (50.0%)	43 (41.0%)
			265	000000000003371	35 (34.0%)			
			190	000000000003731	1 (1.0%)			
			250	000000000000371	1 (1.0%)			
Lineage 4 EURO-AMERICAN	Ural	Ural-1	262	774777777420771	3 (3.0%)	9 (9.0%)	4 (4.0%)	5 (5.0%)
			35	777777777413771	1 (1.0%)			
			1134	777737777420731	1 (1.0%)			
	T	T1	926	777777777760771	1 (1.0%)			
	LAM	LAM8	290	777777400021771	1 (1.0%)			
	Haarlem	H3	50	777777777720771	1 (1.0%)			
			237	777777777700000	1 (1.0%)			

SIT, Spoligotype International Type.

Lineage 2 predominated, accounting for 95 out of 104 strains (91.0%). All Lineage 2 strains belonged exclusively to the Beijing clade. Within this genotype, the SIT1 (Beijing 1, octal code 000000000003771) subtype was the most prevalent, identified in 58 isolates (56.0%), followed by SIT265 (Beijing 265, octal code 000000000003371) in 35 strains (34.0%). The remaining Lineage 2 subtypes, SIT190 and SIT250, were detected in 1 isolate each (1.0%). Epidemiological stratification by patient origin showed that Beijing genotype strains were recovered from 52 (50.0%) foreign nationals and 43 (41.0%) Polish citizens. This corresponded to a Beijing genotype prevalence of 92.9% among foreign nationals (52/56) and 89.6% among Polish patients (43/48), with no statistically significant difference observed between the two groups (p = 0.729, Fisher’s exact test).

Lineage 4 comprised the remaining 9 strains (9.0%), recovered from 5 (5.0%) Polish patients and 4 (4.0%) foreign nationals. Within Lineage 4, the Ural clade was represented by 5 isolates, split into SIT262 (*n* = 3/3.0%), SIT35 (*n* = 1/1.0%), and SIT1134 (*n* = 1/1.0%). The remaining Lineage 4 isolates belonged to the T1 (*n* = 1/1.0%; SIT926), LAM8 (*n* = 1/1.0%; SIT290), and Haarlem families (*n* = 2/2.0%; SIT50 and SIT237).

## Discussion

The study provides the first comprehensive phenotypic and molecular analysis of FQ resistance among *Mycobacterium tuberculosis* strains with MDR and RR isolated between 2022 and 2024.

Particular attention should be paid to the alarmingly high proportion - reaching 43% - of tested strains showing phenotypic resistance to MOX and/or LEV.

As previously described by Kozińska et al., between 2000 and 2009, approximately 14% of FQ-resistant MDR strains were identified in Poland; however, in a subsequent study conducted between 2018 and 2022, this value increased to 40% (Kozińska et al., 2011; Kozińska et al., 2025). The observed further increase in the number of patients with such drug resistance may have important clinical, therapeutic and epidemiological implications. This value significantly exceeds global and regional reports by more than twofold. According to the WHO, in 2023, the proportion of MDR/RR-TB cases with FQ resistance was approximately 18–19% (World Health Organization, 2024). A lower incidence of FQ resistance was also observed in large multicentre analyses. In a meta-analysis covering more than 20 countries, the prevalence of pre-XDR-TB among MDR-TB cases averaged 16–21%, with marked regional variation. Data from Europe typically indicate values of no more than 20–25%, even in countries with a relatively high burden of drug-resistant TB (World Health Organization, 2025). Against this background, the prevalence identified in the present study — more than twice the global average — indicates a critical phenotypic resistance rate within our specific cohort and highlights a particularly unfavorable epidemiological situation in the analyzed population in Poland. However, any direct epidemiological comparison between these datasets must be approached with caution due to fundamental variations in study design, population characteristics, diagnostic methodologies, and sampling frames.

While global and regional surveillance data, such as those reported by the WHO and ECDC, represent aggregated, heterogeneous populations with varying diagnostic capacities, our study focuses on a specific, high-risk cohort of MDR/RR-TB patients in a transitional epidemiological setting. Furthermore, variations in sampling frames, inclusion criteria, and the utilization of highly sensitive diagnostic methods—such as phenotypic drug susceptibility testing combined with advanced molecular sequencing in our study versus standard cultures or incomplete testing in some historical or multi-center cohorts—may influence the direct comparability of these prevalence rates. Despite these inherent methodological differences, the magnitude of the increase observed in our cohort remains a powerful indicator of a shifting resistance landscape.

On an epidemiological level, recent surveillance data confirm that the geopolitical crisis and subsequent population displacement from Ukraine have significantly altered the drug-resistant TB landscape in neighboring Central European countries, including Poland (Dohál et al., 2025). Notably, in our study, 54% of the patient population with FQ-resistant TB were foreigners, primarily of Ukrainian origin. According to some sources, in Ukraine, over one-third of patients in the general population are infected with pre-XDR or XDR strains, and in the southeastern regions, this problem occurs twice as often as in the eastern and central regions (Hogan et al., 2016). Studies monitoring regional hotspots, such as Kharkiv, have documented an explicit and rapid escalation of FQ resistance among drug-resistant isolates, directly reflecting the clinical challenges observed in our study cohort (Konstantynovska et al., 2024).

The observed phenomenon may reflect both the historical therapeutic regimens in the treatment of drug-resistant TB, in which FQs played a central role, and the increasing selection pressure resulting from their long-term and widespread use. This phenomenon has direct implications for the efficacy of the current short MDR/RR-TB treatment regimens recommended by the WHO, based, among others, on MOX.

An important and often underestimated factor contributing to increasing resistance to FQs is their widespread, and often unjustified, use in the treatment of other non-mycobacterial bacterial infections, particularly respiratory and urinary tract infections. Exposure of MTB to FQs prior to a definitive TB diagnosis – often at suboptimal doses and for a short duration – promotes the selection of resistant strains and leads to a delay in the proper diagnosis of the disease. This mechanism has been repeatedly described as one of the key factors in the development of FQ resistance, both in countries with high and low TB incidence (Claeys et al., 2018).

The results also highlight the crucial importance of a rational antibiotic policy (antimicrobial stewardship), which includes limiting the use of FQs outside of clearly defined clinical indications. Numerous studies indicate that uncontrolled use of FQs in the treatment of community-acquired and nosocomial infections significantly contributes to the accumulation of resistance mutations in MTB and increases the risk of pre-XDR-TB forms (Migliori et al., 2012; Claeys et al., 2018). In this context, the WHO and ECDC unequivocally recommend the implementation of rational antibiotic therapy programmes, with a particular focus on limiting the empirical use of FQ in patients with symptoms of TB, until it is unequivocally ruled out (World Health Organization, 2019; European Centre for Disease Prevention and Control and WHO Regional Office for Europe, 2023).

The results emphasise the need for routine early determination of FQ resistance in all MDR/RR-TB patients, which is fundamental for the correct choice of treatment regimen and to limit further transmission of pre-XDR and XDR strains.

In this context, nanopore sequencing presents a highly promising tool for rapid molecular diagnostics. A recent systematic review and meta-analysis demonstrated that this technology provides high diagnostic accuracy for *Mycobacterium tuberculosis* detection (sensitivity ~88.6%, specificity ~93.2%) and, crucially, allows for fast profiling of drug-resistance determinants. Although its accuracy varies depending on the specific drug, integrating nanopore sequencing into routine laboratory practice could significantly accelerate the detection of FQ resistance compared to time-consuming traditional cultures, thereby enabling earlier implementation of targeted treatment (Carandang et al., 2025).

A particularly important observation is that all strains showing phenotypic resistance to FQs were resistant to MOX, and the vast majority of them were also resistant to LEV. This result is of key clinical relevance in the context of the current WHO-recommended treatment regimen for BPaLM, in which an FQ – alongside bedaquiline, pretomanid and linezolid – is one of the pillars of effective therapy (World Health Organization, 2023b).

Molecular analysis revealed that the predominant mechanisms of FQ resistance were mutations within the QRDR of the *gyrA* gene. The most frequently identified alteration was the D94G substitution, followed by D94N and D94A. This aligns with global data underscoring the critical role of *gyrA* codon 94 in conferring high-level phenotypic resistance, which significantly elevates the MICs (Minimum Inhibitory Concentrations) for MOX and LEV (Chien et al., 2016; Uddin et al., 2021). In contrast, less frequent mutations, such as A90V or S91P, are typically associated with lower resistance levels that may occasionally be overcome by FQ dosage escalation. However, cumulative changes or concurrent mutations can still induce a clinically relevant loss of efficacy (Miotto et al., 2018).

From a clinical perspective, the dominance of codon 94 substitutions carries critical therapeutic implications, as their presence signals a definitive loss of FQ efficacy. This directly jeopardizes the success of the ultra-short, highly effective BPaLM regimen recommended by the WHO (World Health Organization, 2023b). In patients harboring these specific *gyrA* variants, MOX can no longer serve as a functional companion drug to protect bedaquiline and pretomanid from the emergence of secondary resistance. Consequently, the detection of any codon 94 substitution renders standard short-course regimens obsolete for this subpopulation, mandating an immediate shift to individualized, longer, or more complex pre-XDR-TB treatment regimens.

The S95T substitution, detected in nearly all analyzed strains, represents a phylogenetic polymorphism rather than a true resistance-conferring mutation. Its widespread presence, particularly among strains belonging to the Beijing lineage, is well-documented in the literature and carries no clinical significance as a resistance marker (Feuerriegel et al., 2014; World Health Organization, 2021). Consequently, this substitution was strictly excluded from subsequent molecular analyses of drug resistance to avoid any overinterpretation of the findings.

True resistance-conferring mutations were primarily located within the QRDR region. Following the exclusion of the S95T polymorphism, the vast majority of resistant strains exhibited single (91% of isolates) or double (3.9% of isolates) genuine resistance mutations in the *gyrA* gene. This strongly confirms the molecular basis of the observed phenotypic resistance and demonstrates high concordance between genetic and phenotypic findings. In contrast, mutations in the *gyrB* gene were sporadic, detected in only 2 isolates, which aligns with reports indicating that *gyrB* alterations serve a complementary role and rarely represent an independent mechanism of FQ resistance.

It is important to highlight that for 5 isolates (5%), no mutations were detected in the *gyrA* and *gyrB* genes. Consequently, it can be hypothesized that an alternative resistance pathway is operating in these strains, or that a coexistence of several distinct mechanisms enhances the resistance phenotype. Among the documented non-target determinants, the overexpression of efflux pump systems is most frequently described. This active transport system is estimated to play a significant role in approximately 30–50% of FQ-resistant strains, with its overexpression in multidrug-resistant strains occurring almost universally as a complementary mechanism supporting target-site point mutations (Mayer and Takiff, 2014; Long et al., 2024). Other described mechanisms involved in mycobacterial resistance to FQs include the protective MfpA (*Mycobacterial fluoroquinolone resistance protein A*) proteins, alterations in cell wall permeability, and mutations located outside the traditionally examined QRDR region that alter gyrase structure and reduce drug affinity (Sayadi et al., 2020).

Nevertheless, it must be emphasized that in the absence of whole-genome sequencing (WGS) or functional validation, these potential explanations remain strictly speculative for the 5 isolates in question, and their definitive resistance mechanisms warrant further investigation.

Deeplex-Myc TB sequencing showed a clear dominance of Lineage 2 and the Beijing clade that characterizes it, comprising more than 90% of FQ-resistant strains in the study group. Beijing strains are commonly associated with a higher ability to acquire drug resistance, increased virulence and more efficient transmission, making them a significant public health risk. The high proportion of Beijing strains in the study population may potentially contribute to both the high prevalence of FQ resistance and the complex multidrug resistance profiles. A strong association between the Beijing genotype and FQ resistance has already been described, confirmed by studies in China and Russia which identified Beijing mycobacteria among 75% and 85% of FQ-resistant isolates, respectively (Duong et al., 2009; Vyazovaya et al., 2023). More recent studies (2016–2021) analyzing LEV and MOX resistance in RIF-resistant strains also reveal a high correlation of the Beijing genotype with FQ resistance, confirming that they are characterized by a higher ability to acquire mutations in the *gyrA* and *gyrB* genes (Nenhan et al., 2025).

It should be noted that in the analysis presented here, 50% of the isolates were Beijing strains cultured from foreigners, mainly from Ukraine, while 41% were Beijing strains isolated from patients of Polish nationality. Between 2000 and 2009, Beijing lineage strains were isolated less frequently among immigrants (41%) and more frequently among Poles (59%) (Kozińska et al., 2011). After 2018, a notable shift in these proportions was observed. In the analysis by Kozińska et al., already in 2018–2022, half of the MDR-TB patient population consisted of foreigners infected with Beijing-TB.

While this massive population migration has drastically increased the absolute burden of FQ-resistant Beijing cases in Poland—with foreign nationals constituting half of our entire study cohort—our comparative analysis revealed no statistically significant difference in Beijing genotype prevalence between foreign and local patients (*p* = 0.729). This critical finding demonstrates that the genetic profile of FQ-resistant TB among Polish nationals has become equally dominated by the Beijing lineage. Consequently, the influx of hyper-endemic strains from across the eastern border has effectively reshaped the entire epidemiological landscape of drug-resistant tuberculosis in Poland, establishing the Beijing genotype as the universal driver of FQ resistance regardless of patient origin.

Nevertheless, it is worth noting that the southern and eastern regions of Ukraine showed an increasing prevalence of the Beijing molecular family, reaching 81% of cases from the Kharkiv region in 2018 (Nikolayevskyy et al., 2007; Kozińska et al., 2025). Therefore, migration remains a plausible explanatory factor rather than a confirmed mechanism for the observed epidemiological shift.

The results emphasize the importance of routine, comprehensive diagnosis of FQ resistance in all patients with suspected MDR/RR-TB, preferably before treatment is initiated. The use of high-resolution molecular methods, such as Deeplex-Myc TB sequencing, enables rapid detection of clinically relevant mutations and allows for rational adjustment of the therapeutic regimen. In the context of the alarming and ubiquitous dominance of FQ-resistant Beijing strains across all patient groups regardless of their origin, these measures take on particular importance for national public health surveillance.

The paper has several limitations that do not allow for a complete assessment of the prevalence of FQ resistance in the TB patient population in Poland. First, although the study cohort represents approximately 80% of all MDR-TB cases in Poland during the study period, the remaining 20% of isolates (*n* = 61) could not be included due to logistic constraints (regional laboratories failing to forward the strains) or technical limitations at the NRLM (such as low bacterial viability). While these missing cases occurred due to random operational factors rather than selective sampling - making a systematic demographic or clinical bias unlikely - their exclusion might still slightly affect the precise estimation of FQ resistance prevalence. A major methodological limitation of this study is the presence of verification bias (partial reference bias), resulting from the exclusive molecular sequencing of the 104 phenotypically fluoroquinolone-resistant isolates. Because the 136 phenotypically susceptible strains were excluded from targeted sequencing, this study cannot evaluate the true diagnostic sensitivity, specificity, positive predictive value, or negative predictive value of QRDR mutations. Consequently, we cannot rule out the potential existence of false-positive, compensatory, or low-level resistance mutations within the unsequenced susceptible population. Furthermore, the study included only MDR-TB cases, without analysis of mono- or FQ-resistant strains among other non-MDR strains, which limits the assessment of the full scale of resistance. Some of the strains included in the analysis had incomplete molecular and phenotypic data, and isolates in which mutations of the QRDR region were not detected in the genome should undergo WGS to detect rare resistance mechanisms. In addition, the variable migration situation during the period under study shifts the demographic proportions within the cohort but makes it difficult to clearly interpret its causal impact on the observed evolutionary trends.

In conclusion, the high proportion of FQ resistance among MliDR/RR strains isolated in Poland between 2022 and 2024, the universal dominance of the Beijing Lineage among both local and foreign-born patients, and the presence of mutations that determine high levels of resistance represent a significant epidemiological and therapeutic challenge. These findings highlight the need for further monitoring of resistance, strengthening molecular surveillance and individualising treatment to effectively control drug-resistant TB in a changing demographic reality.

## Data Availability

The raw data supporting the conclusions of this article will be made available by the authors, without undue reservation.
