# Supplementary material for: Alarming levels of fluoroquinolone resistance among MDR-TB patients in Poland: molecular and phenotypic analysis
Source: Front Cell Infect Microbiol. 2026 Jul 1;16:1881444. doi: 10.3389/fcimb.2026.1881444 (PMC13368571; doi:10.3389/fcimb.2026.1881444)
Supplement: Supplementary file 1 [file Table1.doc]

Table S1. Microbiological and genetic profiles of fluoroquinolone-resistant MDR/RR *Mycobacterium tuberculosis* strains isolated from Polish and foreign-born patients.

| No. | Patient Origin | No.  of strain | Year of isolation | PHENOTYPE | | | | GENOTYPE | | | | | | | | | | SPOLIGOTYPE |
| --- | --- | --- | --- | --- | --- | --- | --- | --- | --- | --- | --- | --- | --- | --- | --- | --- | --- | --- |
|  |  |  |  | LEV | | MOX | | *gyrA* | | | | | | | | *gyrB* | |  |
|  |  |  |  | Sensitive | Resistant | Sensitive | Resistant | G88C | A90V | S91P | D94A | D94G | D94N | D94Y | S95T | N499T | E501V |  |
|  | Polish-born | 198/22 | 2022 |  |  |  |  |  |  |  |  |  |  |  |  |  |  | Beijing 1 |
|  | Foreign-born | 101 | 2022 |  |  |  |  |  |  |  |  |  |  |  |  |  |  | Beijing 1 |
|  | Polish-born | 92/22 | 2022 |  |  |  |  |  |  |  |  |  |  |  |  |  |  | Beijing 265 |
|  | Polish-born | 98/22 | 2022 | no data | no data |  |  |  |  |  |  |  |  |  |  |  |  | Beijing 265 |
|  | Polish-born | 236/22 | 2022 |  |  |  |  |  |  |  |  |  |  |  |  |  |  | Beijing 1 |
|  | Polish-born | 424/22 | 2022 | no data | no data |  |  |  |  |  |  |  |  |  |  |  |  | Beijing 1 |
|  | Polish-born | 70/22 | 2022 | no data | no data |  |  |  |  |  |  |  |  |  |  |  |  | Ural-1 262 |
|  | Polish-born | 118/22 | 2022 | no data | no data |  |  |  |  |  |  |  |  |  |  |  |  | Beijing 1 |
|  | Foreign-born | 223/22 | 2022 |  |  |  |  |  |  |  |  |  |  |  |  |  |  | Ural-1 1134 |
|  | Polish-born | 25/22 | 2022 | no data | no data |  |  |  |  |  |  |  |  |  |  |  |  | H3 237 |
|  | Polish-born | 135/22 | 2022 | no data | no data |  |  |  |  |  |  |  |  |  |  |  |  | Beijing 265 |
|  | Polish-born | 35/22 | 2022 | no data | no data |  |  |  |  |  |  |  |  |  |  |  |  | Beijing 265 |
|  | Foreign-born | 222/22 | 2022 |  |  |  |  |  |  |  |  |  |  |  |  |  |  | Ural-1 262 |
|  | Polish-born | 123/22 | 2022 | no data | no data |  |  |  |  |  |  |  |  |  |  |  |  | Beijing 1 |
|  | Polish-born | 175/22 | 2022 |  |  |  |  |  |  |  |  |  |  |  |  |  |  | Beijing 265 |
|  | Foreign-born | 159/22 | 2022 | no data | no data |  |  |  |  |  |  |  |  |  |  |  |  | Beijing 1 |
|  | Foreign-born | 119/22 | 2022 | no data | no data |  |  |  |  |  |  |  |  |  |  |  |  | Beijing 1 |
|  | Foreign-born | 136/22 | 2022 |  |  |  |  |  |  |  |  |  |  |  |  |  |  | Beijing 1 |
|  | Polish-born | 239/22 | 2022 |  |  |  |  |  |  |  |  |  |  |  |  |  |  | Beijing 1 |
|  | Foreign-born | 94/22 | 2022 | no data | no data |  |  |  |  |  |  |  |  |  |  |  |  | Beijing 1 |
|  | Foreign-born | 233/22 | 2022 |  |  |  |  |  |  |  |  |  |  |  |  |  |  | Beijing 265 |
|  | Polish-born | 150/22 | 2022 | no data | no data |  |  |  |  |  |  |  |  |  |  |  |  | Beijing 1 |
|  | Polish-born | 49/22 | 2022 | no data | no data |  |  |  |  |  |  |  |  |  |  |  |  | Beijing 265 |
|  | Foreign-born | 96 | 2022 | no data | no data |  |  |  |  |  |  |  |  |  |  |  |  | LAM8 290 |
|  | Polish-born | 12/22 | 2022 | no data | no data |  |  |  |  |  |  |  |  |  |  |  |  | Beijing 1 |
|  | Polish-born | 15/22 | 2022 | no data | no data |  |  |  |  |  |  |  |  |  |  |  |  | Beijing 1 |
|  | Polish-born | 43/22 | 2022 | no data | no data |  |  |  |  |  |  |  |  |  |  |  |  | Beijing 1 |
|  | Polish-born | 91/22 | 2022 | no data | no data |  |  |  |  |  |  |  |  |  |  |  |  | Beijing 265 |
|  | Foreign-born | 92/23 | 2023 |  |  |  |  |  |  |  |  |  |  |  |  |  |  | Beijing 1 |
|  | Foreign-born | 601/23 | 2023 |  |  |  |  |  |  |  |  |  |  |  |  |  |  | Beijing 1 |
|  | Polish-born | 7606 | 2023 |  |  |  |  |  |  |  |  |  |  |  |  |  |  | Beijing 265 |
|  | Polish-born | 1753 | 2023 |  |  |  |  |  |  |  |  |  |  |  |  |  |  | Beijing 265 |
|  | Polish-born | 2551 | 2023 |  |  |  |  |  |  |  |  |  |  |  |  |  |  | Beijing 265 |
|  | Foreign-born | 400/23 | 2023 |  |  |  |  |  |  |  |  |  |  |  |  |  |  | Beijing 265 |
|  | Foreign-born | 351/23 | 2023 |  |  |  |  |  |  |  |  |  |  |  |  |  |  | Beijing 1 |
|  | Foreign-born | 192/23 | 2023 |  |  |  |  |  |  |  |  |  |  |  |  |  |  | Beijing 190 |
|  | Foreign-born | 2854 | 2023 |  |  |  |  |  |  |  |  |  |  |  |  |  |  | Beijing 1 |
|  | Foreign-born | 5333 | 2023 |  |  |  |  |  |  |  |  |  |  |  |  |  |  | Beijing 1 |
|  | Foreign-born | 442/23 | 2023 |  |  |  |  |  |  |  |  |  |  |  |  |  |  | Beijing 1 |
|  | Polish-born | 2374/23 | 2023 |  |  |  |  |  |  |  |  |  |  |  |  |  |  | Beijing 265 |
|  | Polish-born | 107/23 | 2023 |  |  |  |  |  |  |  |  |  |  |  |  |  |  | Beijing 265 |
|  | Foreign-born | 401/23 | 2023 |  |  |  |  |  |  |  |  |  |  |  |  |  |  | Beijing 265 |
|  | Foreign-born | 441/23 | 2023 |  |  |  |  |  |  |  |  |  |  |  |  |  |  | Beijing 1 |
|  | Polish-born | 79/23 | 2023 |  |  |  |  |  |  |  |  |  |  |  |  |  |  | Beijing 1 |
|  | Foreign-born | 437/23 | 2023 |  |  |  |  |  |  |  |  |  |  |  |  |  |  | Beijing 1 |
|  | Polish-born | 140/23 | 2023 |  |  |  |  |  |  |  |  |  |  |  |  |  |  | Beijing 265 |
|  | Foreign-born | 128/23 | 2023 |  |  |  |  |  |  |  |  |  |  |  |  |  |  | Beijing 1 |
|  | Polish-born | 462/23 | 2023 |  |  |  |  |  |  |  |  |  |  |  |  |  |  | Beijing 265 |
|  | Foreign-born | 142/23 | 2023 |  |  |  |  |  |  |  |  |  |  |  |  |  |  | Beijing 265 |
|  | Polish-born | 1205 | 2023 |  |  |  |  |  |  |  |  |  |  |  |  |  |  | Beijing 1 |
|  | Foreign-born | 7172 | 2023 |  |  |  |  |  |  |  |  |  |  |  |  |  |  | Beijing 1 |
|  | Foreign-born | 4124 | 2023 |  |  |  |  |  |  |  |  |  |  |  |  |  |  | Beijing 1 |
|  | Foreign-born | 337/23 | 2023 |  |  |  |  |  |  |  |  |  |  |  |  |  |  | Beijing 265 |
|  | Foreign-born | 346/23 | 2023 |  |  |  |  |  |  |  |  |  |  |  |  |  |  | Beijing 265 |
|  | Foreign-born | 310/23 | 2023 |  |  |  |  |  |  |  |  |  |  |  |  |  |  | Beijing 265 |
|  | Polish-born | 352/23 | 2023 |  |  |  |  |  |  |  |  |  |  |  |  |  |  | Beijing 1 |
|  | Polish-born | 163 | 2023 |  |  |  |  |  |  |  |  |  |  |  |  |  |  | Beijing 1 |
|  | Foreign-born | 295/23 | 2023 |  |  |  |  |  |  |  |  |  |  |  |  |  |  | Beijing 265 |
|  | Polish-born | 429/23 | 2023 |  |  |  |  |  |  |  |  |  |  |  |  |  |  | Ural-1 262 |
|  | Foreign-born | 49/23 | 2023 |  |  |  |  |  |  |  |  |  |  |  |  |  |  | Beijing 1 |
|  | Polish-born | 143/23 | 2023 |  |  |  |  |  |  |  |  |  |  |  |  |  |  | Beijing 265 |
|  | Polish-born | 415/23 | 2023 |  |  |  |  |  |  |  |  |  |  |  |  |  |  | Beijing 1 |
|  | Foreign-born | 37/23 | 2023 |  |  |  |  |  |  |  |  |  |  |  |  |  |  | Beijing 1 |
|  | Foreign-born | 71/23 | 2023 |  |  |  |  |  |  |  |  |  |  |  |  |  |  | Beijing 1 |
|  | Foreign-born | 98/23 | 2023 |  |  |  |  |  |  |  |  |  |  |  |  |  |  | Beijing 1 |
|  | Polish-born | 108/23 | 2023 |  |  |  |  |  |  |  |  |  |  |  |  |  |  | Beijing 265 |
|  | Foreign-born | 314/23 | 2023 |  |  |  |  |  |  |  |  |  |  |  |  |  |  | Beijing 1 |
|  | Polish-born | 1778 | 2023 |  |  |  |  |  |  |  |  |  |  |  |  |  |  | Beijing 265 |
|  | Foreign-born | 99/23 | 2023 |  |  |  |  |  |  |  |  |  |  |  |  |  |  | Beijing 1 |
|  | Foreign-born | 235/24 | 2024 |  |  |  |  |  |  |  |  |  |  |  |  |  |  | Beijing 265 |
|  | Foreign-born | 108/24 | 2024 |  |  |  |  |  |  |  |  |  |  |  |  |  |  | Beijing 1 |
|  | Foreign-born | K09 | 2024 |  |  |  |  |  |  |  |  |  |  |  |  |  |  | Beijing 265 |
|  | Polish-born | 244/24 | 2024 |  |  |  |  |  |  |  |  |  |  |  |  |  |  | Beijing 1 |
|  | Polish-born | 145/24 | 2024 |  |  |  |  |  |  |  |  |  |  |  |  |  |  | Ural-1 35 |
|  | Foreign-born | 4/24 | 2024 |  |  |  |  |  |  |  |  |  |  |  |  |  |  | Beijing 1 |
|  | Foreign-born | 222 | 2024 |  |  |  |  |  |  |  |  |  |  |  |  |  |  | T1 926 |
|  | Polish-born | 142/24 | 2024 |  |  |  |  |  |  |  |  |  |  |  |  |  |  | Beijing 1 |
|  | Foreign-born | 141/24 | 2024 |  |  |  |  |  |  |  |  |  |  |  |  |  |  | Beijing 1 |
|  | Foreign-born | 201/24 | 2024 |  |  |  |  |  |  |  |  |  |  |  |  |  |  | Beijing 1 |
|  | Polish-born | 29/24 | 2024 |  |  |  |  |  |  |  |  |  |  |  |  |  |  | Beijing 1 |
|  | Foreign-born | 144/24 | 2024 |  |  |  |  |  |  |  |  |  |  |  |  |  |  | Beijing 1 |
|  | Polish-born | 76/24 | 2024 |  |  |  |  |  |  |  |  |  |  |  |  |  |  | Beijing 265 |
|  | Polish-born | 333/24 | 2024 |  |  |  |  |  |  |  |  |  |  |  |  |  |  | H3 50 |
|  | Foreign-born | 237/24 | 2024 |  |  |  |  |  |  |  |  |  |  |  |  |  |  | Beijing 1 |
|  | Foreign-born | 2099 | 2024 |  |  |  |  |  |  |  |  |  |  |  |  |  |  | Beijing 1 |
|  | Foreign-born | 223/24 | 2024 |  |  |  |  |  |  |  |  |  |  |  |  |  |  | Beijing 265 |
|  | Foreign-born | 1/24 | 2024 |  |  |  |  |  |  |  |  |  |  |  |  |  |  | Beijing 265 |
|  | Foreign-born | 11/24 | 2024 |  |  |  |  |  |  |  |  |  |  |  |  |  |  | Beijing 1 |
|  | Polish-born | 183/24 | 2024 |  |  |  |  |  |  |  |  |  |  |  |  |  |  | Beijing 265 |
|  | Polish-born | 138/24 | 2024 |  |  |  |  |  |  |  |  |  |  |  |  |  |  | Beijing 1 |
|  | Foreign-born | 129 | 2024 |  |  |  |  |  |  |  |  |  |  |  |  |  |  | Beijing 1 |
|  | Polish-born | 105/24 | 2024 |  |  |  |  |  |  |  |  |  |  |  |  |  |  | Beijing 265 |
|  | Polish-born | 7168 | 2024 |  |  |  |  |  |  |  |  |  |  |  |  |  |  | Beijing 1 |
|  | Foreign-born | 315/24 | 2024 |  |  |  |  |  |  |  |  |  |  |  |  |  |  | Beijing 265 |
|  | Foreign-born | 8234 | 2024 |  |  |  |  |  |  |  |  |  |  |  |  |  |  | Beijing 265 |
|  | Polish-born | 30348 | 2024 |  |  |  |  |  |  |  |  |  |  |  |  |  |  | Beijing 265 |
|  | Foreign-born | 376/24 | 2024 |  |  |  |  |  |  |  |  |  |  |  |  |  |  | Beijing 1 |
|  | Foreign-born | 8315 | 2024 |  |  |  |  |  |  |  |  |  |  |  |  |  |  | Beijing 1 |
|  | Foreign-born | 982 | 2024 |  |  |  |  |  |  |  |  |  |  |  |  |  |  | Beijing 1 |
|  | Foreign-born | 38/24 | 2024 |  |  |  |  |  |  |  |  |  |  |  |  |  |  | Beijing 1 |
|  | Foreign-born | 369/24 | 2024 |  |  |  |  |  |  |  |  |  |  |  |  |  |  | Beijing 1 |
|  | Foreign-born | 408/24 | 2024 |  |  |  |  |  |  |  |  |  |  |  |  |  |  | Beijing 1 |
|  | Polish-born | 200/24 | 2024 |  |  |  |  |  |  |  |  |  |  |  |  |  |  | Beijing 250 |
|  | Polish-born | 337/24 | 2024 |  |  |  |  |  |  |  |  |  |  |  |  |  |  | Beijing 1 |

*Abbreviations:* **LEV**, levofloxacin; **MOX**, moxifloxacin; **QRDR**, quinolone resistance-determining region; **MDR/RR-TB**, multidrug-resistant/rifampicin-resistant tuberculosis.
Note: *Data from 18 isolates from 2022 are missing for the LEV phenotype due to diagnostic protocol changes.
